# Exploring Teachers’ Satisfaction and Students’ Entrepreneurial Competencies in Four Entrepreneurial Programs Carried Out in Extremadura (Spain) Schools

**DOI:** 10.3389/fpsyg.2020.484103

**Published:** 2021-01-14

**Authors:** Juan José Maldonado Briegas, Antonio Citarella, Ana Isabel Sánchez Iglesias, Sergio Gonzáles Ballester, A. Javier Alvarez Marínez, Florencio Vicente Castro

**Affiliations:** ^1^Department of Economics, University of Extremadura, Badajoz, Spain; ^2^Department of Health Science, University of Burgos, Burgos, Spain; ^3^Department of Health Sciences, Miguel de Cervantes European University, Valladolid, Spain; ^4^INFAD Researcher, Badajoz, Spain

**Keywords:** teachers’ well-being, entrepreneurial culture, entrepreneurship, entrepreneurial education, entrepreneurial programs

## Abstract

The question of whether the entrepreneur is born or made, forces us to respond that the entrepreneurial culture clearly breaks with the myth that entrepreneurs are born. Currently, it is considered that entrepreneurial skills can be acquired like any other discipline and that is why it should be taught (Timmons, 2003). Their teaching and learning are key to the well-being of the teacher and to the positive achievement of the students. The entrepreneurial culture is an educational objective of developed societies and has its origin in the business culture (Peña Calvo et al., 2015). There are two current models, American and European (Erkkilä, 2000). The one that develops in the Extremadura region (Spain), based on the European model, tries to create a vital state in the participating students that enhances competences such as creativity, personal initiative or self-confidence, elements that try to generate a true motivation to undertake. The motivated, accomplished and positive teacher contributes to that achievement and achieving it favors the well-being of teachers. It’s a round trip. It is a “do ut des.” In our research, we evaluated four entrepreneurial culture programs in the classroom: “Junioremprende,” “Teenemprende,” “Experemprende,” and “Youthemprende.” The teachers involved in the programs are 356, and the valid questionnaires are 154. The students enrolled in the programs are 4800, and the questionnaires valid 1198. In the questionnaire, one of the key questions is “general teacher satisfaction,” with 17 common questions for all the programs. An essential question posed is: “I am satisfied with the achievements obtained by my students to participate in the program” The results of our research, according to a seven-point Likert scale, gives a very high degree of teachers’ satisfaction. Their satisfaction was centered on the perception of their teachings are conducive to the achievement of his students. The degree of teacher satisfaction for each of the programs and significantly means differences were found with Junior program resulted the more satisfied for teachers. For students, participation in the programs resulted in high development of entrepreneurial competencies. Similarly to teachers results, Junior program was found more significant in developing students’ entrepreneurial competencies indicating that entrepreneurial education is very appropriate for children. Findings of this study suggest that entrepreneurial education should be encouraged for students at every stage of education.

## Introduction

Project-based learning are commonly considered a successful methodology allowing students to acquire XXI century skills which include a number of several soft skills required in the workplace. Actively participating in their own social environment and cooperating in group in order to solve a problem, students are engaged in a proactive construction of their learning in which they experiment also a more effective interaction with their teachers-facilitators. For this purpose, different typologies of project in which students actively participate in their social environment are usually carried out at every stage of educational curriculum worldwide. Among these, projects-based learning focusing on entrepreneurial skills and competencies are interesting even more educational institution due to the growing importance to stimulate self-employment and business creation.

In an ideal perspective, the progress and development of society have historically been linked to visionary men and women that caused a change of mentality within the established order. These individuals possessed certain attitudes that made them venture into disruptive projects with the society in which they lived. These people who agglutinate attitudes such as creative destruction, creation of value, identification of opportunities and ingenuity are those currently known as entrepreneurs. In this regard, Cubico et al. (2010) noted that in the last decades psychological research on entrepreneurs has found” that certain individual differences (i.e., traits, ability, attitude, cognition, values motives, goals.) are seen that distinguish between entrepreneurs and other people. Moreover, these personal qualities are present in different measures in successful entrepreneurs than in unsuccessful ones” (p. 426). Thus, a number of individual factors interact with organizational and environmental factors in order to promote entrepreneurial outcomes as –for example – new venture success (Baum and Locke, 2004). The broad space of individual qualities includes several entrepreneurial skills and competencies which were increasingly considered like something that can acquired through training. Therefore, entrepreneurial education (EE), programs of EE in university and school, and their efficacy in promoting entrepreneurship received a growing academic interest across worldwide in the last decades (Ronstadt, 1985; Jones and English, 2004; Pihie and Bagheri, 2010; Elmuti et al., 2012; Elert et al., 2015; Oehler et al., 2015; Hahn et al., 2017; Cho and Lee, 2018). The key concept of entrepreneurial education rooted in the current assumption that entrepreneur is “made” through experience acquired throughout life and not “born,” hence, “through effective entrepreneurship education an individual can access the skills and knowledge needed to start and grow up a new business” (Raposo and Do Paço, 2011, p. 454). Accordingly, several competencies can be identified for entrepreneurship and growing corpora of programs worldwide aim to teach them to students involved in formal and informal education.

In European context, entrepreneurship is currently considered a key factor for economic growth and a specific framework – the *Entrepreneurship Competence Framework* (EntreComp) (Bacigalupo et al., 2016) – was developed for teaching entrepreneurial competencies within of programs of EE at schools, university and for lifelong learning. Bacigalupo et al. (2016) claim that “EntreComp defines entrepreneurship as a transversal competence, which applies to all spheres of life: from nurturing personal development, to actively participating in society, to (re)entering the job market as an employe or as a self-employed person, and also to starting up ventures (cultural, social or commercial)” (p. 6). In the EntreComp model were identified two main dimensions including three tightly intertwined competence areas, namely “Ideas and Opportunity” “Resources” “Into Action,” containing the following 15 competences:

1. Ideas and Opportunity:
  • Spotting opportunities
  • Creativity
  • Vision
  • Valuing ideas
  • Ethical and sustainable thinking
2. Resources:
  • Self-awareness and self-efficacy
  • Motivation and perseverance
  • Mobilizing resource
  • Financial and economic literacy
  • Mobilizing others
3. Into Action:
  • Learning through experience
  • Working with other
  • Coping with ambiguity, uncertainty and risk
  • Planning and management
  • Taking the initiative

These competencies will be result in entrepreneurial learning outcomes at progressive level of proficiency after the completing actions of education training programs developed both in formal and informal educational context.

EntreComp, hence, provide to national educational systems a comprehensive theoretical framework to insert entrepreneurial education in curricula. Naturally, countries independently choose how entrepreneurial courses are carried out in schools, university and informal training. This produce a different picture of entrepreneurial education among European countries. For example, Cubico et al. (2015) identify the University of Wismar as a best practice of transversally integrated entrepreneurial education in university curricula in order to promote entrepreneurial intentions also in students who never considered entrepreneurship as career path. Diversely, analyzing data from Italian university students, authors highlighted the Italian delay in response to European indication for entrepreneurial education probably determined by the lack of a national strategic plan, unless through technical and vocational courses.

Principally, entrepreneurial programs are developed in higher education where is possible to evaluate the immediate subsequent impact of the entrepreneurial education in start-up of new business and entrepreneurial intention. Therefore, study on programs of entrepreneurial teaching reporting mostly finding for this level of education. For example, Lüthje and Franke (2002) analyzing entrepreneurial intentions and they evaluation of entrepreneurial education programs in their university of German students compared to students of MIT of Boston. Results showed that German students have a significantly lower intention to start a business after leaving university than Boston students. In addition, students of MIT evaluate to a greater extent than German students that their university entrepreneurial education in terms of providing skills and knowledge and perceived their university as active in promoting future entrepreneurs in pre-creation stage. Hence, authors conclude that United States entrepreneurial programs can represented as a success model for academic institution in German. This example shows that in European context entrepreneurial education need to stronger its impact in order to stimulate the European desiderate goal of promoting entrepreneurship and self-employ. Whilst, as abovementioned, entrepreneurial programs principally involved students of higher education, even more educational systems at national level promote the development of entrepreneurial programs at earlier level. In this regard, Toutain and Fayolle (2017) claim that entrepreneurial competences should be taught at even-early age and “the success of XXI century education relies on the capacity of schools and vocational training centers to help learners acquire entrepreneurial competencies, which require them to take control and self-direct their own learning process” (p. 3). For this purpose, an important role will be played by teachers, and a central issue relies in their capacity to adopt a teaching even more centered on autonomy, optimism, self-directed learners ‘construction of knowledge and skills, academic achievement and positive teacher-student relationship that, in turn, also promote a better psychological well-being for teachers (Spilt et al., 2011; Roffey, 2012; Kamil, 2014). Entrepreneurial education with its focus on competences aiming to stimulate self-determined teaching and learning and proactive behavior can be a tool for fostering both students and teacher satisfaction, well-being and positive and society valued outcomes. In this regard, Niemiec and Ryan (2009) stressed that “people are innately curious, interested creatures who possess a natural love of learning and who desire to internalize the knowledge, customs, and values that surround them”(p.133). Therefore, students’ entrepreneurial competencies and entrepreneurial teaching may be strongly linked with self-determination theory in education (Niemiec and Ryan, 2009) as they could improve desire of knowledge and autonomy in acquire it. In explaining motivational process, Self-determination focuses on three basic psychological needs: competence, relatedness and autonomy. Especially, autonomy results a central construct for understanding links with self-determinate teaching and learning in entrepreneurial education. Indeed, autonomy often explains the main motivation and satisfaction for entrepreneurship and in educational setting both teaching and learning should be centered in reinforcing and promoting it (van Gelderen, 2010). In addition, autonomy links entrepreneurial education also with self-directed learning theory stressing the autonomous process in which “individuals select, manage, and assess their own learning activities, which can be pursued at any time, in any place, through any means, at any age” (van Gelderen, 2010 p.8).

Based on these theoretical assumptions, this study aims to explore teacher satisfaction with entrepreneurial education and entrepreneurial competences in students ranging from primary schools to high school. Four programs developed in Extremadura (Spain) were evaluated using self-reported teachers’ and students’ data. The principal descriptive objective consists in assessing the level of teacher satisfaction and students entrepreneurial learning outcomes for each program developed and eventual differences among them. Moreover, we aim to assess if significant differences exist for programs by considering age of participants. We strongly believed that educational programs on entrepreneurship can be really appreciated by teachers as stimulating their supportive-autonomy teaching and promoting entrepreneurial competencies in students also in early stage of schooling.

## Programs of Entrepreneurial Culture in Extremadura

Education and training in Extremadura in the field of entrepreneurship is carried out within Extremadura regional policy, through the Education Act of Extremadura 4/2011, of March 7, where it considers priority aspects in the curriculum emotional intelligence and the entrepreneurial capacity as transversal axes of education, seeking to promote, among other aspects, the entrepreneurial and creative capacity in the region. This strategy for the promotion of entrepreneurial capacity is aligned with the strategic lines established by the EU in 2002 to promote entrepreneurship and entrepreneurship in the primary and secondary education system, through the report of the group of experts “Education and training in entrepreneurship”; and that in 2008, the Commission of the European Communities established in the publication in the Small Business Act the importance of fostering the entrepreneurial culture and facilitating the exchange of good practices in education for entrepreneurship. In this way, the Extremaduran government through the Order of May 24 (DOE no 64 of June 5, 2007) has regulated aspects related to the organization and implementation of the Mandatory Secondary Education taught by the Organic Law 2006, of May 3, Education, among them, the obligation to offer a subject related to the entrepreneurial initiative in the teaching of Compulsory Secondary Education also specifying content, objectives and evaluation criteria in each educational stage. Optional subjects related to the promotion of the entrepreneurial culture in each and every one of the educational levels have also been included in the curriculum and different permanent training programs for teachers have been defined in relation to these values, thereby promoting entrepreneurial initiatives in educational centers. Extremadura promotes entrepreneurial culture through regulated education offering an itinerary with various actions throughout the curriculum, from primary education to university, and through different programs. Among the programs offered are:

JuniorEmprende: an educational program where the entrepreneurial culture is fostered in the 5th and 6th grade students, and where they work as a team, through project-based learning, developing a project during a full academic year, making the ideas translate into actions. In this program creativity, autonomy and teamwork skills are increased. In addition, the teaching staff is accompanied both inside and outside the classroom, thus allowing to create a network of teachers interested in the entrepreneurial culture.

ExpertEmprende: is a program that tries to promote and promote the initiative and entrepreneurial culture in the students of FP of Middle and Higher Grade, through the creation of business projects with possibilities of development in Extremadura. This program seeks to bring students to the business world seeking to strengthen knowledge, attitudes and skills related to entrepreneurship that have been worked in previous stages and in turn encourages the approach to the business world, both students and faculty, through the design and development of a business plan. The objectives are directly linked to the entrepreneurial venture. It is intended to involve students in the design and development of a business plan.

YouthEmprende: program aimed at high school students with the aim of discovering and working on entrepreneurial skills related to the search for solutions to social and economic challenges in the environment.

TeenEmprende: is a program that aims to promote entrepreneurial culture in students of Compulsory Secondary Education and Vocational Training (FP), working entrepreneurial skills of students, such as leadership, teamwork, and conflict management. The purpose is the active participation and involvement of students in their social environment through the development of a project that allows networking with the team of participating teachers.

## Materials and Methods

### Participants and Procedure

*356* teachers were involved in the programs with total valid questionnaires n. 154. The students enrolled in the programs were 4800, and the questionnaires valid 1198. Authors choose to exclude questionnaires with missing data and valid scale must be considered as items totally completed by respondents. All participants work and study in schools from Extremadura region in Spain. Data were collected in classroom during about 1 h. No ethic approval for this study was necessary, following the national Spanish rules of the psychologist association as well as the Ministry of the Education. This study is a part of a larger project aiming to analyze various aspects of teacher well-being in their workplace. For participating to this general project an informed consent was requested to participants.

### Measures

As part of a larger project, measures were extrapolated to others scales and structured as *ad hoc* scales for the specific purposes of this study.

In order to assess teachers’ satisfaction with the programs an *ad hoc* scale (see Appendix) was developed by researcher. The measure is composed by 17 items and each question was evaluated on a 7-point Likert scale, ranging from dissatisfaction to total satisfaction. Principal Component Analysis was performed to validate the scale. Results indicated a one-dimension factorial structure with all factor-loading values above 0.40. A Cronbach Alpha coefficient of 0.842 indicated a good reliability of the scale.

Four *ad hoc* scales (see Appendix) were developed to assess students’ entrepreneurial competencies, namely Personal Skills, Process and Results Competences, Intellectual Competences and Social Competences. Personal Skills measure is composed by 10 items each question was evaluated on a 7-point Likert scale, ranging from totally disagree to totally agree. The scale contains 5 indicators: (1) Motivation and personal initiative; (2) Vision; (3) Self- Confidence; (4) Autonomy; and (5) Flexibility. Principal Component Analysis was performed to validate the scale. Results indicated a one-dimension structure with all factor-loading values above 0.40. A Cronbach Alpha coefficient of 0.844 indicated a good reliability of the scale. Process and Results Competencies measure is composed by 11 items and each question was evaluated on a 7-point Likert scale, ranging from totally disagree to totally agree. The scale contains 5 indicators: (1) Work Capacity; (2) Planning; (3) Commitment; (4) Quality; and (5) Social Responsibility. Principal Component Analysis was performed to validate the scale. Results indicated a one-dimension structure with all factor-loading values above 0.40. A Cronbach Alpha coefficient of 0.862 indicated a good reliability of the scale. Intellectual Competencies measure is composed by 10 items and each question was evaluated on a 7-point Likert scale, ranging from totally disagree to totally agree. The scale contains 5 indicators: (1) Exploratory Capacity; (2) Creativity; (3) Innovation; (4) Ability to identify and solve problems; and (5) Self-learning. Principal Component Analysis was performed to validate the scale. Results indicated a one-dimension structure with all factor-loading values above 0.40. A Cronbach Alpha coefficient of 0.857 indicated a good reliability of the scale. Social Competencies measure is composed by 12 items and each question was evaluated on a 7-point Likert scale, ranging from totally disagree to totally agree. The scale contains 5 indicators: (1) Business Orientation; (2) Communication; (3) Teamwork (4) Empathy and Self-awareness; and (5) Leadership (5). Principal Component Analysis was performed to validate the scale. Results indicated a one-dimension structure with all factor-loading values above 0.40. A Cronbach Alpha coefficient of 0.861 indicated a good reliability of the scale.

## Results

In order to evaluate teachers’ satisfaction and students’ competencies in each program descriptive statistics, Anova and *post hoc* tests were performed using SPSS 21 software. Significant Kolmogorov–Smirnov and Shapiro–Wilk test indicated non-normality of data collected. However, this result should not invalidate our findings because existing evidences showed that Anova is a robust test when assumption of normality is violated (e.g., Blanca et al., 2017).

Table 1 shows means for each item of the questionnaire concerning teachers’ satisfaction with all four programs. The highest total value was reached by the first item “The program is an opportunity to work explicitly important skills for the development of students” while the lowest was reached by item 9 “The training I received as a teacher has been sufficient for the implementation of the program.” Therefore, teachers generally stressed the importance of entrepreneurial programs for their students but refer that they need to improve their competences to better implement it since they didn’t receive a sufficient training. The total value of item 16 (5.96) “I am satisfied with the achievements obtained by my group (s) for participating in the program” denoted an overall satisfaction with the programs also reinforced by the high value of item 17 (6.14) “I intend to participate in forthcoming editions of the Entrepreneurial Culture programs.”

**TABLE 1:** Means of teachers’ satisfaction for each item of the questionnaire in the four programs.

**Item**	**Junior Emprende**	**Expert Emprende**	**Youth Emprende**	**Teen Emprende**	**Total**
1	6.47	6.31	6.25	6.26	6.32
2	6.09	6.10	6.12	5.85	6.04
3	5.86	5.60	5.66	5.61	5.68
4	6.08	5.94	5.73	5.91	5.92
5	5.51	5.53	5.62	5.37	5.51
6	5.43	5.45	5.38	5.24	5.38
7	6.10	6.08	6.16	6.11	6.11
8	6.19	5.90	5.97	5.93	6.00
9	5.77	5.30	5.21	5.26	5.38
10	6.05	5.56	5.61	5.44	5.67
11	5.81	5.47	5.45	5.22	5.49
12	5.88	5.79	5.82	5.42	5.73
13	5.64	5.51	5.47	5.20	5.46
14	6.09	5.75	5.73	5.60	5.79
15	6.23	6.08	6.01	5.88	6.05
16	6.36	5.95	5.93	5.60	5.96
17	6.32	6.08	6.09	6.07	6.14

Table 2 shows descriptive statistics, Anova and *post hoc* test results for teachers’ satisfaction among the four programs. We can observe that means revealed a general teachers’ satisfaction for all programs. However, Anova results *F(3,612)* = 8.41 *p* = *0.00* indicated a significantly means differences among the four programs. Specifically, *post hoc* test showed that teachers have been significantly more satisfied in Junior programs compared to other three programs.

**TABLE 2:** Teachers’ satisfaction among four programs (Anova and *post hoc*).

**Teacher satisfaction**	**Program**	***N***	***Mean***	***SD***	**ANOVA**		
					***F***	**Sig.**	
	Junior (g1)	154	5.99	0.629	8.41	0.000	Means differences (*post hoc*) ∣g1–g2∣* ∣g1–g3∣* ∣g1–g4∣* ∣g2–g3∣∣g2–g4∣∣g3–g4∣
	Expert (g2)	154	5.79	0.611			
	Youth (g3)	154	5.78	0.556			
	Teen (g4)	154	5.64	0.656			

*Significant means difference at 0.05 level.

Table 3 shows descriptive statistics, Anova and *post hoc* test results for all indicators of self-reported students’ Personal skills for the four programs. We can observe that means revealed a good general level for all programs. However, results of Anova and *post hoc* test indicated significantly means differences among the four programs. A significantly higher level of Motivation and Vision self-reported students involved in Junior Program compared to the other three program. Students of Junior Program also self-reported a significantly higher level of Self-confidence, Autonomy and Flexibility than students of Teen Program. Students of Expert Program self-reported a significantly higher level of Vision and Self-confidence compared to students of Teen Program. Finally, students of Youth Program self-reported a significantly higher level of Vision, Self-confidence and Autonomy compared to students of Teen Program.

**TABLE 3:** Students’ personal skills among four programs (Anova and *post hoc*).

Motivation	**Program**	***N***	***Mean***	***SD***	**ANOVA**		
					***F***	**Sig.**	
	Junior (g1)	328	5.72	1.10	11.93	0.00	Means differences (*post hoc*) ∣g1–g2∣* ∣g1–g3∣* ∣g1–g4∣* ∣g2–g3∣∣g2–g4∣∣g3–g4∣
	Expert (g2)	330	5.32	1.04			
	Youth (g3)	254	5.39	1.06			
	Teen (g4)	286	5.25	1.08			
	Total	1198	5.43	1.09			
Vision	Junior (g1)	328	5.83	1.08	12.23	0.00	Means differences (*post hoc*)
	Expert (g2)	330	5.56	1.05			∣g1–g2∣* ∣g1–g3∣* ∣g1–g4∣* ∣g2–g3∣∣g2–g4∣*∣g3–g4∣*
	Youth (g3)	254	5.59	0.974			
	Teen (g4)	286	5.32	1.08			
	Total	1198	5.58	1.06			
Self-confidence	Junior (g1)	328	5.90	1.07	20.33	0.00	Means differences (*post hoc*)
	Expert (g2)	330	5.70	1.08			∣g1–g2∣∣g1–g3∣∣g1–g4∣* ∣g2–g3∣∣g2–g4∣*∣g3–g4∣*
	Youth (g3)	254	5.75	0.966			
	Teen (g4)	286	5.26	1.06			
	Total	1198	5.66	1.07			
Autonomy	Junior (g1)	328	5.66	1.08	4.43	0.00	Means differences (*post hoc*)
	Expert(g2)	330	5.53	1.05			∣g1–g2∣∣g1–g3∣∣g1–g4∣* ∣g2–g3∣∣g2–g4∣∣g3–g4∣*
	Youth (g3)	254	5.60	1.02			
	Teen (g4)	286	5.36	1.13			
	Total	1198	5.54	1.08			
Flexibility	Junior (g1)	328	5.71	1.07	4.82	0.00	Means differences (*post hoc*)
	Expert (g2)	330	5.33	1.20			∣g1–g2∣∣g1–g3∣∣g1–g4∣* ∣g2–g3∣∣g2–g4∣∣g3–g4∣
	Youth (g3)	254	5.48	2.18			
	Teen (g4)	286	5.35	1.21			
	Total	1198	5.47	1.43			

*Significant means difference at 0.05 level.

Table 4 shows descriptive statistics, Anova and *post hoc* test results for all indicators of self-reported students’ Process and Results Competences for the four programs. Means revealed a good general level for all programs. Anova and *post hoc* test indicated significant means differences among the four programs for the five indicators of Process and Results Competences. A significantly higher level of Work Capacity self-reported students involved in Junior Program compared to the other three programs. Students of Junior Program also self-reported a significantly higher level of Planning, Commitment, Quality and Social Responsibility than students of Teen Program. Students of Expert Program self-reported a significantly lower level of Quality compared to Junior Program students and a significantly higher level of Work Capacity, Planning, and Social Responsibility compared to students of Teen Program. Finally, students of Youth Program self-reported a significantly higher level of Quality than students of Youth Program.

**TABLE 4:** Students’ process and results competencies among four programs (Anova and *post hoc*).

**Work capacity**	**Program**	***N***	***Mean***	***SD***	**ANOVA**		
					***F***	**Sig.**	
	Junior (g1)	328	6.14	0.921	13.67	0.00	Means differences (*post hoc*) ∣g1–g2∣* ∣g1–g3∣* ∣g1–g4∣* ∣g2–g3∣∣g2–g4∣*∣g3–g4∣
	Expert (g2)	330	5.91	1.02			
	Youth (g3)	254	5.84	1.09			
	Teen (g4)	286	5.60	1.16			
	Total	1198	5.89	1.06			
Planning	Junior (g1)	328	5.86	1.07	6.22	0.00	Means differences (*post hoc*) ∣g1–g2∣∣g1–g3∣∣g1–g4∣* ∣g2–g3∣∣g2–g4∣*∣g3–g4∣
	Expert (g2)	330	5.76	1.04			
	Youth (g3)	254	5.71	0.972			
	Teen (g4)	286	5.49	1.19			
	Total	1198	5.71	1.08			
Commitment	Junior (g1)	328	5.81	1.05	3.96	0.01	Means differences (*post hoc*) ∣g1–g2∣∣g1–g3∣∣g1–g4∣* ∣g2–g3∣∣g2–g4∣∣g3–g4∣
	Expert (g2)	330	5.73	1.08			
	Youth (g3)	254	5.72	1.01			
	Teen (g4)	286	5.52	1.16			
	Total	1198	5.70	1.08			
Quality	Junior (g1)	328	6.08	0.905	10.54	0.00	Means differences (*post hoc*) ∣g1–g2∣* ∣g1–g3∣∣g1–g4∣* ∣g2–g3∣∣g2–g4∣∣g3–g4∣*
	Expert (g2)	330	5.83	0.973			
	Youth (g3)	254	5.89	0.896			
	Teen (g4)	286	5.63	1.16			
	Total	1198	5.86	1.002			
Social responsibility	Junior (g1)	328	5.88	0.959	4.60	0.00	Means differences (*post hoc*) ∣g1–g2∣∣g1–g3∣∣g1–g4∣* ∣g2–g3∣∣g2–g4∣*∣g3–g4∣
	Expert (g2)	330	5.86	1.02			
	Youth (g3)	254	5.79	1.09			
	Teen (g4)	286	5.60	1.17			
	Total	1198	5.79	1.06			

*Significant means difference at 0.05 level.

Table 5 shows descriptive statistics, Anova and *post hoc* test results for all indicators of self-reported students’ Intellectual Competences for the four programs. Means revealed a good general level for all programs. Anova and *post hoc* test results indicated significant means differences among the four programs. A significantly higher level of Exploratory Capacity, Creativity, Innovation, Ability to identify and solve problems and Self-learning self-reported students involved in Junior Program compared to students of the other three programs. Students of Expert Program self-reported a significantly higher level of Work Capacity, Innovation and Ability to identify and solve problems compared to students of Teen Programs. Finally, students of Youth Program self-reported a significantly higher level of Innovation than students of Teen Program.

**TABLE 5:** Students’ intellectual competences among four programs (Anova and *post hoc*).

**Exploratory capacity**	**Program**	***N***	***Mean***	***SD***	**ANOVA**		
					***F***	**Sig.**	
	Junior (g1)	328	6.20	0.882	27.97	0.00	Means differences (*post hoc*) ∣g1–g2∣* ∣g1–g3∣* ∣g1–g4∣* ∣g2–g3∣∣g2–g4∣*∣g3–g4∣*
	Expert (g2)	330	5.74	1.13			
	Youth (g3)	254	5.70	0.928			
	Teen (g4)	286	5.46	1.16			
	Total	1198	5.79	1.07			
Creativity	Junior (g1)	328	5.95	0.945	13.43	0.00	Means differences (*post hoc*) ∣g1–g2∣* ∣g1–g3∣* ∣g1–g4∣* ∣g2–g3∣∣g2–g4∣∣g3–g4∣
	Expert (g2)	330	5.62	1.12			
	Youth (g3)	254	5.57	0.961			
	Teen (g4)	286	5.43	1.16			
	Total	1198	5.65	1.07			
Innovation	Junior (g1)	328	6.04	0.960	14.86	0.00	Means differences (*post hoc*) ∣g1–g2*∣∣g1–g3∣* ∣g1–g4∣* ∣g2–g3∣∣g2–g4∣*∣g3–g4∣*
	Expert (g2)	330	5.80	1.07			
	Youth (g3)	254	5.75	0.878			
	Teen (g4)	286	5.49	1.19			
	Total	1198	5.78	1.05			
Ability to identify and solve problems	Junior (g1)	328	6.22	0.853	15.44	0.00	Means differences (*post hoc*) ∣g1–g2∣* ∣g1–g3∣* ∣g1–g4∣* ∣g2–g3∣∣g2–g4∣*∣g3–g4∣
	Expert (g2)	330	5.94	1.02			
	Youth (g3)	254	5.86	1.03			
	Teen (g4)	286	5.67	1.17			
	Total	1198	5.94	1.04			
Self-learning	Junior (g1)	328	6.04	0.952	7.36	0.00	Means differences (*post hoc*) ∣g1–g2∣* ∣g1–g3∣* ∣g1–g4∣* ∣g2–g3∣∣g2–g4∣∣g3–g4∣
	Expert(g2)	330	5.80	1.14			
	Youth (g3)	254	5.79	0.966			
	Teen (g4)	286	5.64	1.15			
	Total	1198	5.83	1.07			

*Significant means difference at 0.05 level.

Table 6 shows descriptive statistics, Anova and *post hoc* test results for all indicators of self-reported students’ Social Competences for the four programs. Means revealed a good general level for all programs. Anova and *post hoc* test indicated significant means differences among the four programs. A significantly higher level of Communication, Teamwork and Empathy and Self-awareness self-reported students involved in Junior Program compared to the other three program. Students of Junior Program also self-reported a significantly higher level of Business Orientation and Leadership compared to students of Youth and Teen Program. Students of Expert Program self-reported a significantly higher level of Business orientation compared to students of Teen Program.

**TABLE 6:** Students’ social competences among four programs (Anova and *post hoc*).

Business orientation	**Program**	***N***	***Mean***	***SD***	**ANOVA**		
					***F***	**Sig.**	
	Junior (g1)	328	6.12	0.875	11.1	0.00	Means differences (*post hoc*) ∣g1–g2∣∣g1–g3∣* ∣g1–g4∣* ∣g2–g3∣∣g2–g4∣*∣g3–g4∣
	Expert (g2)	330	5.94	0.928			
	Youth (g3)	254	5.88	0.883			
	Teen (g4)	286	5.68	1.14			
	Total	1198	5.92	0.972			
Communication	Junior (g1)	328	6.19	0.902	8.32	0.00	Means differences (*post hoc*) ∣g1–g2∣* ∣g1–g3∣* ∣g1–g4∣* ∣g2–g3∣∣g2–g4∣∣g3–g4∣
	Expert (g2)	330	5.89	1.02			
	Youth (g3)	254	5.87	1.11			
	Teen (g4)	286	5.80	1.15			
	Total	1198	5.95	1.05			
Teamwork	Junior (g1)	328	6.19	0.869	10.66	0.00	Means differences (*post hoc*) ∣g1–g2*∣∣g1–g3∣* ∣g1–g4∣* ∣g2–g3∣∣g2–g4∣∣g3–g4∣
	Expert (g2)	330	5.96	1.01			
	Youth (g3)	254	5.94	1.004			
	Teen (g4)	286	5.73	1.13			
	Total	1198	5.96	1.14			
Empathy and self-awareness	Junior (g1)	328	6.21	0.842	9.43	0.00	Means differences (*post hoc*) ∣g1–g2∣* ∣g1–g3∣* ∣g1–g4∣* ∣g2–g3∣∣g2–g4∣∣g3–g4∣
	Expert(g2)	330	5.95	0.987			
	Youth (g3)	254	5.91	0.855			
	Teen (g4)	286	5.82	1.14			
	Total	1198	5.98	0.974			
Leadership	Junior (g1)	328	6.16	0.894	6.09	0.00	Means differences (*post hoc*) ∣g1–g2∣∣g1–g3∣* ∣g1–g4∣* ∣g2–g3∣∣g2–g4∣∣g3–g4∣
	Expert (g2)	330	5.98	1.02			
	Youth (g3)	254	5.91	0.867			
	Teen (g4)	286	5.84	1.10			
	Total	1198	5.98	0.981			

*Significant means difference at 0.05 level.

Finally, Table 7 shows descriptive statistics, Anova and *post hoc* test results for the overall measured four axis of skills and competences among the for programs. Anova and *post hoc* test results indicated significant means differences among the four programs. A significantly higher level Intellectual and Social Competences self-reported students involved in Junior Program compared to the other three programs. Students of Junior Program also self-reported a significantly higher level of Personal Skills compared to students of Expert Program and a significantly higher level of Process and Results Competences than Students of Teen Program. Students of Expert Program self-reported a significantly higher level of Process and Results Competences compared to students of Teen Programs while, finally, students of Youth Program self-reported a significantly higher of Personal Skills and Process and Result Competences compared to students of Teen Program.

**TABLE 7:** Students’ overall skills and competences among four programs (Anova and *post hoc*).

Personal skills	**Program**	***N***	***Mean***	***SD***	**ANOVA**		

					***F***	**Sig.**	
	Junior (g1)	328	5.76	1.04	10.95	0.00	Means differences (*post hoc*) ∣g1–g2∣* ∣g1–g3∣∣g1–g4∣* ∣g2–g3∣∣g2–g4∣∣g3–g4∣*
	Expert (g2)	330	5.49	0.953			
	Youth (g3)	254	5.56	1.01			
	Teen (g4)	286	5.31	1.02			
	Total	1198	5.54	1.02			
Process and results competences	Junior (g1)	328	5.95	0.907	7.81	0.00	Means differences (*post hoc*) ∣g1–g2∣∣g1–g3∣∣g1–g4∣* ∣g2–g3∣∣g2–g4∣*∣g3–g4∣*
	Expert (g2)	330	5.82	0.951			
	Youth (g3)	254	5.79	0.848			
	Teen (g4)	286	5.57	1.15			
	Total	1198	5.78	0.981			
Intellectual competences	Junior (g1)	328	6.09	0.845	17.56	0.00	Means differences (*post hoc*) ∣g1–g2∣* ∣g1–g3∣* ∣g1–g4∣* ∣g2–g3∣∣g2–g4∣*∣g3–g4∣
	Expert (g2)	330	5.78	1.01			
	Youth (g3)	254	5.73	0.807			
	Teen (g4)	286	5.54	1.13			
	Total	1198	5.80	0.979			
Social competences	Junior (g1)	328	6.17	0.815	10.19	0.00	Means differences (*post hoc*) ∣g1–g2∣* ∣g1–g3∣* ∣g1–g4∣* ∣g2–g3∣∣g2–g4∣∣g3–g4∣
	Expert (g2)	330	5.95	0.912			
	Youth (g3)	254	5.90	0.860			
	Teen (g4)	286	5.76	1.10			
	Total	1198	5.95	0.935			

*Significant means difference at 0.05 level.

## Discussion

This study aimed to explore the level of teacher satisfaction with entrepreneurial education and the entrepreneurial competencies of students involved in four entrepreneurial programs carried out in schools of Extremadura (Spain). Results indicated that the medium level of teacher satisfaction is high for all programs. Similarly, the level of self-reported four dimensions of entrepreneurial competencies acquired by students resulted high in the four programs. However, significant differences were found between the four programs both for teachers’ satisfaction and students’ competencies. Specifically, the Junior Emprende program resulted in stronger teacher satisfaction and students’ competencies while Teen Emprende program was significantly weaker for these outcomes.

The better results found for Junior program suggest that entrepreneurial education is very appreciated by students at earlier stage of education that, in turn, affect also teachers’ satisfaction. In this regard, some findings in literature investigating entrepreneurial education for children confirmed our results and suggest a number of several reasons explaining them. Indeed, despite research focusing on programs of entrepreneurial education for children is quite rare in literature (Pereira et al., 2007), findings of some studies indicated the effectiveness of them in promoting various positive outcomes. For instance, Rosendahl Huber et al. (2014), in their randomized field experiment, evaluated the effect of *BizWorld* program *–* a very internationally spread entrepreneurial education program for primary school – on the entrepreneurial cognitive and non-cognitive skills in Dutch children. They found that with exception of *persistence* for all non-cognitive skills the difference between treatment group and control group was significantly positive. Instead, the difference in cognitive entrepreneurial skills was not significant. In addition, the study evaluated also children’ entrepreneurial intention to start a business in the future which resulted significantly decreasing for children in treatment group. However, authors recommend to consider this surprising finding with caution “due to the lack of validated measure of entrepreneurial intentions for children” (p. 18). In conclusion, authors stressed the importance of the development of entrepreneurial skills in earlier age and that it might be a better investment compared doing them in adolescence because these effects could represent a positive outcome in the long run in terms of solid base competence for subsequent entrepreneurial education in high school or university. Agreeing with Rosendahl Huber et al. (2014), a question particularly important could be the following: Why entrepreneurial education may be more attractive for younger people? Maybe, a possible answer could be that younger people are naturally enjoying in activity stimulating experiential learning and playing. For this reason, Löbler (2006) highlighted how constructivist perspective may represent the better teaching approach in order to develop entrepreneurial competences in children. In fact, formal education based on transmission of knowledge and traditional assessment – still present in many European schools – probably mortify the children natural propensity to creativity, teamwork and learning by doing resulting –among others – as very important entrepreneurial characteristics and entrepreneurial spirit. With its focus on students independent building of knowledge, constructivist approach allows to improve entrepreneurial competences in a funny and not boring manner. It’s clear that teachers have a fundamental role in this process. They must agree that entrepreneurial education may be really effective in this perspective building a learning environment and classroom climate that stimulate the full expression of children ideas. Even more, hence, entrepreneurial programs – as general education – are inspired – or they should be inspired – by constructivist perspective. Consequently, also teachers’ satisfaction could be improved in this process, as demonstrated by results of our study where programs developed were informed by experiential learning. Finally, another important reason underlying attractiveness of entrepreneurial education for younger people could be found in the even more massive use of technological tools in many programs. In this regard, the three – dimension virtual world has been identified as a potential effective tool in order to support entrepreneurial education due to power of it to create learning situation in which children simulate at small-scale business operations (Pereira et al., 2018). Therefore, literature shows that the more advanced process of learning in entrepreneurial education perfectly embraces the currently widely validated teaching approach, namely constructivist perspective and ICT use in education, in order to prepare children and adolescents to successful compete in the labor-market after completion of school and university.

This study has several limitations. The main concerns the research design eminently descriptive. Indeed, in order to properly evaluated the effective significant impact of the four programs on students’ competencies the elective research design should have been the experimental or quasi-experimental methodology. Additionally, also for teachers’ satisfaction the impact of programs would have been more scientifically significant if other variables were analyzed or controlled. In particular, some professional characteristics as specific subject taught or teaching style could be influent in determining satisfaction in entrepreneurial programs. Another several limitation concerns instruments used to collect data. Indeed, existing validated instruments with better established would be more useful and powerful in measuring constructs instead of *ad hoc* scales used in this study. Despite this, this limitation partially was justified by the fact of structuring two instruments strictly adapted to the evaluation of specific educational entrepreneurial programs carried out in Extremadura schools.

Despite these limitations, our study can suggest several implications for practice and stimulate future research. It seems clear, in fact, that entrepreneurial education is largely appreciated in school context and particularly at early stage of education. The fact that teacher and students were positive involved with the programs indicate that they can be replicated and improved in Extremadura educational system, in Spain and, in general, in Europe. Our study contributes to support the effectiveness of entrepreneurial education and previous findings in this regard. For example, in Spanish context Hernández-Sánchez et al. (2019) evaluated the relationship between programs of entrepreneurial education and the rating of entrepreneurial activity in a number of regions in Spain. Conclusions show the real effectiveness of entrepreneurial programs for this purpose and suggest a number of implications for practice including the necessity to change entrepreneurial subject taught in classroom from optional to compulsory. In this regard, Penaluna et al. (2020) stressed the pioneering work of North Macedonia and Wales in introducing a compulsory Entrepreneurship and Innovation curriculum in their school system. Authors define North Macedonian and Welsh educationalist as the European “frontiersmen in entrepreneurial education” (p. 259). Another important aspect could be stressed considering findings of our study. Indeed, developing efficacious and appreciated entrepreneurial programs at early stage of education, could have a positive impact on career trajectories of youths often less involved in traditional school subjects. In same case, these students would be interested to remain in the educational circuit if their practical thinking was valued in learning activities as entrepreneurship simulation. Therefore, a double positive effect could be obtained for these students: (1) if they have an instinctive vocation for entrepreneurship they can find in school and university a place where cultivate their passion acquiring professional skills indispensables for business in knowledge society; and (2) entrepreneurial education can serve to indirect instruments to stimulate passion for study in general. Finally, for all students, we don’t forget that entrepreneurial education at every stage of education can be the coming out of unsuspected entrepreneurial talents which only attends to be discovered and supported. In this regard, importantly, Krüger and David (2020) by developing an inclusive entrepreneurial education framework for person with disability stressed the importance of entrepreneurial education in promoting inclusion and social innovation. Therefore, the initiatives of the promotion of entrepreneurship in the classrooms carried out by the administration are seen as a bet for the future and a long-term investment in a wide range of educational practices. Learning to undertake favors better educational results and can translate into greater employability, greater work well-being and higher quality of life in adulthood for all youths. For this purpose, it is never too early to develop certain behaviors, skills and abilities.

## Data Availability Statement

The datasets generated for this study are available on request to the corresponding author.

## Author Contributions

JM and AC realized the literature review and statistical analysis of the article. AS realized the theoretical framing of the article in the research fields of psychology and education. SG realized the graphic design of tables. AA and FV realized the final version of the article. All authors contributed to the article and approved the submitted version.

## Conflict of Interest

The authors declare that the research was conducted in the absence of any commercial or financial relationships that could be construed as a potential conflict of interest.

## APPENDIX

*Ad Hoc* Questionnaires

Teachers’ satisfaction scale

S1 The program is an opportunity to work explicitly important skills for the development of students.

S2 The Involvement of the center’s management has allowed an optimal development of the activities put into operation.

S3 The timing of the program allows a good development of the activities to be carried out.

S4 The type of activities and methodology proposed make it easier to work on the objectives proposed by the program.

S5 I have used the work proposal offered by the technical team.

S6 The program has been executed transversally, working at some time each of the key competences.

S7 The technical attention received by the organization of the program has been sufficient.

S8 The resources offered on the Entrepreneurial Culture website of the Junta de Extremadura are useful for the execution of the program.

S9 The training I received as a teacher has been sufficient for the implementation of the program.

S10 I have had the opportunity to get in touch with teachers to share doubts and knowledge that have enriched me as an education professional.

S11 The involvement of the students has favored an execution of the activities at an optimum level.

S12 The project has improved the active participation of students in the classroom.

S13 The implementation of the program favors the inclusion of students in situations of risk and with special educational needs.

S14 The work done by the students has brought them closer to their surroundings, allowing them to know their needs and opportunities.

S15 In general, we have had the necessary support for the correct development of the program.

S16 I am satisfied with the achievements obtained by my groups for participating in the program.

S17 I intend to participate in future editions of the Entrepreneurial Culture programs.

Personal Skills scale

Motivation and personal initiative:

• I value the possibility of applying my ideas and developing my work as I like it, without depending on the decisions of other people;
• When I have a personal project or an initiative I find a way to carry it out.

Vision:

• I believe that situations of change in life are always opportunities to improve;
• I have the ability to understand each new context, anticipate situations and even create them.

Self-confidence:

• I pose ideas and solutions to problems, although they may be different than expected;
• I’d rather be wrong than do nothing.

Autonomy:

• I value independence and be able to do what I like;
• I am a decisive person and capable of making complex or hasty decisions.

Flexibility:

• When something does not go as I expected, I do not get discouraged, and I try again in another way and as many times as necessary;

I cope well the risk and uncertainty.

Process and Results Competences scale

Work capacity:

• I finish what I start whenever I can or my abilities allow me;
• In a situation of hard work I try to do my best to finish on time.

Planning:

• When I have to perform a new task I try to be clear about the objective and try to be aware of what it means (in time, cost, resources.) to organize it as best as possible;
• In a situation of overwork I think it is good to share tasks and responsibilities, even if that means losing a bit of control.

Commitment:

• I am able of taking responsibility;
• When the task to do affects the team, I prefer that responsibility be shared among all;
• When I have to do something that I do not like I try to do it as soon as possible.

Quality:

• I’m always looking to improve everything I do;
• Doing things with quality leads to feelings of satisfaction.

Social responsibility:

• In everything I do I try to balance my good and that of others;
• The school is not only a place where to develop personally and professionally, but also to improve the environment and improve the world in which we live.

Intellectual Competences scale

Exploratory capacity:

• I like the challenge of doing something new;
• Before starting a new task I try to inform myself and document everything I can before doing it.

Creativity:

• When I have a problem I try to reformulate it until I find one or several ways to solve it;
• I like to follow my intuition, although sometimes it leads me to seemingly irrational or meaningless approaches.

Innovation:

• I like to try new ways of doing things, even if the traditional way is good;
• Having information is important to anticipate and plan for the future.

Ability to identify and solve problems

• When I have to deal with a problem, I analyze it first and then I focus on the search for solutions;
• Having information is important to anticipate and plan for the future.

Self-learning:

• When I face with an error, I try to analyze the situation and learn from the error;
• I worry about learning when I am interested in a specific subject and consider it important for me.

Social Competences scale

Business orientation:

• It’s nice to work in a group even with colleagues that I do not know previously because I like to meet new people;
• When I work in a team I try to observe and learn from all the members;
• When necessary, I can be a persuasive and convincing person;
• I like to make friends and have a wide social network with which to maintain contact.

Communication:

• When I get new information or knowledge, I share it with the team;
• When I have to explain something to the team, I am quite efficient and it does not cost me to make myself understood.

Teamwork:

• I like to work in a team and I’m good at it;
• I have initiative and I participate actively in the teams coordinating the group.

Empathy and self-awareness:

• I try to get to know the people of the team to understand their opinions and reactions and to contribute to everything going well;
• I am aware of my emotions and I control my moods so as not to spoil my – relationships with others.

Leadership:

• It is usual for team members to seek my opinion because I have the ability to transmit my energy and motivate others to achieve the team’s objectives;
• When someone has talent, I realize, value them and help them contribute as much as possible with their ideas.
